# Physical Activity Engagement After Tai Ji Quan Intervention Among Older Adults With Mild Cognitive Impairment or Memory Concerns: A Secondary Analysis of a Randomized Clinical Trial

**DOI:** 10.1001/jamanetworkopen.2024.50457

**Published:** 2024-12-17

**Authors:** Fuzhong Li, Peter Harmer, Elizabeth Eckstrom, Kerri Winters-Stone

**Affiliations:** 1 Oregon Research Institute, Springfield; 2 Division of General Internal Medicine & Geriatrics, Oregon Health & Science University, Portland; 3 Knight Cancer Institute, Oregon Health & Science University, Portland

## Abstract

**Importance:**

The effect of exercise interventions on increasing and sustaining moderate to vigorous physical activity (MVPA) among older adults with mild cognitive impairment (MCI), who are at heightened risk of dementia, remains unclear.

**Objective:**

To examine whether participation in a 6-month, supervised, home-based tai ji quan intervention increases MVPA among US community-dwelling older adults at 1 year.

**Design, Setting, and Participants:**

This study was a secondary analysis of a parallel-group, outcome assessor–blinded, randomized clinical trial conducted virtually at participants’ homes. Eligible participants were aged 65 years or older, lived independently, and had a baseline Clinical Dementia Rating (CDR) global score of 0.5 or less. Participants were enrolled between October 1, 2019, and June 30, 2022. Final follow-up occurred in May 2023.

**Interventions:**

Participants were randomly assigned (1:1:1) to standard tai ji quan, cognitively enhanced tai ji quan, or stretching, and they exercised (via real-time videoconferencing) 1 hour semiweekly for 6 months.

**Main Outcomes and Measures:**

The primary outcome was self-reported time engaged in MVPA (in minutes per week), assessed with the International Physical Activity Questionnaire at baseline and at 4, 6, and 12 months following randomization. The Physical Activity Guidelines for Americans recommend that older adults achieve at least 150 min/wk of MVPA, and the number of participants meeting this recommendation was also assessed. Analyses followed the intention-to-treat principle.

**Results:**

Among the 318 participants enrolled, 107 were randomized to standard tai ji quan, 105 to cognitively enhanced tai ji quan, and 106 to stretching. Their mean (SD) age was 76 (5) years, 212 (66.7%) were women, and 247 (77.7%) had a CDR global score of 0.5. A total of 304 participants (95.6%) completed the trial, and 299 (94.0%) had complete data on the primary outcome at the 12-month follow-up. At 12 months, both tai ji quan groups had increased MVPA levels compared with the stretching control group, with mean differences from baseline of 66 min/wk (95% CI, 25-108 min/wk; *P* = .002) with standard tai ji quan and 65 min/wk (95% CI, 24-108 min/wk; *P* = .002) with cognitively enhanced tai ji quan. Odds ratios for meeting the recommended amount of MVPA (≥150 min/wk) were 3.11 (95% CI, 1.75-5.53; *P* < .001) for the standard tai ji quan group and 3.67 (95% CI, 2.02-6.65; *P* < .001) for the cognitively enhanced tai ji quan group compared with the stretching group.

**Conclusion and Relevance:**

In this secondary analysis of a randomized clinical trial involving older adults with MCI or self-reported memory concerns, home-based tai ji quan training (delivered via videoconferencing) increased MVPA 6 months following cessation of the intervention compared with stretching. These findings suggest that tai ji quan may be promoted as an avenue to achieve physical activity guidelines for older adults with MCI or subjective memory concerns.

**Trial Registration:**

ClinicalTrials.gov Identifier: NCT04070703

## Key Points

**Question:**

Can a 6-month, supervised tai ji quan intervention increase and sustain physical activity among older adults with mild cognitive impairment (MCI) or self-reported memory concerns after cessation of the intervention?

**Findings:**

In this secondary analysis of a randomized clinical trial of 318 participants, older adults (aged ≥65 years) who performed home-based standard or cognitively enhanced tai ji quan (delivered via videoconferencing) increased moderate to vigorous intensity physical activity (MVPA) by a mean of 65 min/wk at 12 months compared with those assigned to a stretching control. Tai ji quan participants were also more likely to achieve the recommended 150 min/wk of MVPA at 12 months.

**Meaning:**

These findings support the inclusion of tai ji quan as a means to promote physical activity among community-dwelling older adults with MCI or memory concerns.

## Teaser

This secondary analysis of a randomized clinical trial explores the effect of a 6-month home-based tai ji quan intervention on engagement in moderate to vigorous physical activity at 12 months among US community-dwelling older adults with mild cognitive impairment (MCI) or self-reported memory concerns.

## Introduction

Current evidence indicates that exercise interventions delivered by group-based, home-based, or digital approaches produce major health benefits^1,2,3,4^ and promote engagement in physical activity (PA) among healthy older adults.^5,6,7^ Evidence of the effectiveness of these interventions for individuals with subjective memory complaints or mild cognitive impairment (MCI) is less clear.^8^ More than half of older adults with cognitive impairment are either physically inactive or insufficiently active.^9,10^ Older adults with MCI exhibit lower levels of PA and greater sedentary behavior than those without MCI,^11^ and they are 9 times more likely than those most active to develop incident dementia.^12^ Because physical inactivity is associated with numerous negative health consequences,^13,14^ particularly a higher risk of cognitive decline^15^ and development of dementia,^16^ there is a critical public health need to promote PA to prevent dementia and Alzheimer disease^17,18^ in this at-risk population.

Tai ji quan, a therapeutic mind-body exercise, produces multiple health benefits for older adults,^19^ including cognitive health in those with MCI,^20^ and is recommended for older adults to increase their levels of PA.^21^ Previous studies have shown that community-living older adults who completed structured tai ji quan interventions continued with tai ji quan, sought out other community-based activities, or both.^22,23,24^ These results suggest that tai ji quan may be uniquely qualified to engage more community-dwelling older adults with MCI in free-living PA (ie, self-initiated PA that is performed at one’s own pace and in one’s own environment). In addition, tai ji quan requires minimal space and no special equipment, and it has shown promise as a low-cost,^25,26^ cognitively enhancing,^20^ safe,^20,27^ and scalable modality^22,23,28,29,30,31^; these features are clinically and pragmatically well suited for this at-risk population.

This study examined whether participation in a 6-month virtual tai ji quan–based intervention, compared with a stretching exercise control, would result in increased moderate to vigorous intensity PA (MVPA) measured at 6 months after termination of the structured intervention in older adults with MCI or memory concerns. We hypothesized that tai ji quan training, relative to stretching, would increase free-living MVPA in those with MCI or memory concerns at postintervention follow-up. In an exploratory analysis, we examined change in intensity-specific PA (ie, moderate intensity, vigorous intensity) and movement confidence between tai ji quan and stretching and the proportion of tai ji quan participants, relative to those in the stretching group, who met the recommended level of MVPA (≥150 min/wk) per the Physical Activity Guidelines for Americans (hereinafter, the MVPA guideline).^21^

## Methods

### Study Design and Setting

This is a secondary analysis of a parallel-group, outcome assessor–blinded, randomized clinical trial. Details of the trial design and protocol have been described elsewhere^20^ and are summarized in Supplement 1. The trial was approved by the Oregon Research Institute Institutional Review Board and overseen by a safety officer appointed by the National Institute on Aging. Written informed consent was obtained from all participants. This study followed the Consolidated Standards of Reporting Trials (CONSORT) reporting guideline.

The trial was conducted with community-dwelling adults living independently in homes across rural, suburban, and urban communities in the US. The interventions were delivered primarily to participants via real-time videoconferencing. After completing the 6-month active intervention, participants were followed for 6 months post intervention, during which no structured activities were prescribed to them.

Study recruitment lasted from October 1, 2019, to June 30, 2022. Follow-up visits ended in May 2023.

### Study Participants

Eligible participants aged 65 years or older met the following criteria: (1) had self-reported (by the participant and an informant) memory decline or memory concerns, (2) had a Clinical Dementia Rating (CDR)^32^ global score of 0.5 or less, (3) had no clinical diagnosis of dementia or substantial cognitive impairment (score of ≥24 on the Mini-Mental State Examination [MMSE]^33^), and (4) had no major difficulties in performing activities of daily living. We excluded individuals who had participated in structured rigorous activities or muscle-strengthening more than 2 times a week for more than 15 minutes per session in the previous 3 months, showed symptoms of substantial depression (score >4 on the Geriatric Depression Scale^34^), were unable to commit to the full intervention or unwilling to accept group assignment, or lacked medical clearance.

### Recruitment

Recruitment methods mainly involved media campaigns via social media, mass mailings to the target population, and word of mouth. Research staff contacted individuals who responded to the trial promotion and confirmed the initial eligibility criteria. A separate interview was conducted with an informant who provided information regarding the respondent’s cognitive status based in part on a CDR assessment. Individuals were further evaluated, via an online face-to-face videoconference session, on additional eligibility criteria, including CDR and MMSE scores. Those who met the eligibility criteria and signed the study consent form underwent an online baseline assessment.

### Randomization and Intervention

Participants were randomly assigned to receive 1 of the 3 interventions (standard tai ji quan, cognitively enhanced tai ji quan, or stretching exercise) in a 1:1:1 allocation via a computer-generated (with random block lengths) randomization sequence. The allocation sequence was concealed to investigators, interventionists, and participants. Study assessors were masked to group allocation. Participants were not masked to intervention assignment.

All 3 intervention groups participated in 1 hour of training twice weekly for 24 weeks.^20^ The standard tai ji quan intervention involved repeated practices of forms and minitherapeutic exercises designed for training limits of stability, postural control, balance recovery, and sensory integration and compensation,^20,27^ including (1) postural alignment, (2) symmetrical movements, (3) controlled displacement of the body’s center of mass over the base of support, (4) coordinated eyes-head-hand movements during motion, (5) multidirectional stepping, (6) rotational ankle sway, and (7) self-initiated reactive postural responses.

Cognitively enhanced tai ji quan concomitantly added a set of cognitive exercises involving interactive, reactive, and verbal and nonverbal cueing. Exercises required participants to (1) verbally repeat, after the interventionist, the step-by-step movements in a form; (2) respond to or practice the intended form while inhibiting or suppressing unintended movement; (3) exercise recall of forms; (4) practice associating form names with form numbers; (5) perform forms without verbal or visual instructional cues; (6) perform side switching, switch forms when given out of sequence, and vary form sequences; and (7) perform forms with word spelling.^20^

The stretching control involved whole-body stretching exercises.^20,27^ Specific activities included both general and region-specific upper-body and lower-extremity stretches, musculoskeletal joint movements, and comfortable abdominal breathing exercises, with exercises performed in alternation between a seated or standing position.

### Follow-Up

Participants were followed for 6 months after the 6-month structured and supervised online exercise classes stopped.^20^ During this time, participants were encouraged to stay physically active and were provided with recordings of completed classes and other online exercise resources. Participants then reported, through a survey, their continued exercise frequency and duration at the 12-month assessment follow-up.

### Measurement and Outcomes

All participants were assessed at baseline, 4 months, 6 months (intervention termination), and 12 months (postintervention follow-up). Participants completed a health survey that included a PA questionnaire at each assessment time point, via a secured online link access to the Qualtrics system (qualtrics.com). Other measurements, including body weight, height, and blood pressure, were obtained at baseline through a videoconference session via self-reports or assessments (when a device was available). Demographic characteristics, such as age, sex, race and ethnicity, and education level, were also obtained via the survey. Race and ethnicity were self-reported as Asian, Black or African American (hereinafter, Black), Hispanic, White, or other race or ethnicity (including American Indian or Alaska Native or Native Hawaiian or Other Pacific Islander); these data were collected to describe the study population.

The primary outcome was weekly MVPA assessed using the International Physical Activity Questionnaire–Short Form (IPAQ-S).^35^ Participants reported how often (in days) and how much time (in minutes) over the previous week they participated in PA or exercise of moderate intensity (gardening, cleaning, bicycling at a regular pace, swimming, or other fitness activities) (3-5.9 metabolic equivalents of task [METs]) or vigorous intensity (heavy lifting, heavy gardening, construction work, chopping wood, aerobics, jogging or running, fast bicycling, or other activities) (≥6 METs) that lasted at least 10 minutes. These activities did not include the time spent participating in the assigned exercise interventions in the study. The total weekly duration of MVPA, measured in minutes, was calculated by multiplying frequency times duration (vigorous activity was weighted by 2) to form a total amount of MVPA in minutes per week.

The secondary measures were moderate intensity PA (in minutes per week), vigorous intensity PA (in minutes per week), meeting the recommended MVPA guideline (≥150 min/wk),^21^ and movement confidence. PA measures were derived from the IPAQ-S, and movement confidence was measured with the Activities-Specific Balance Confidence (ABC) scale.^36^ The ABC scale assesses one’s confidence in performing various daily activities (eg, picking up an object from the floor, standing on a chair to reach for something, or walking on icy sidewalks) without compromising one’s balance. Items on the ABC measure were rated by participants on a scale from 0% (no confidence) to 100% (complete confidence), and the total score was calculated by averaging the scores from all 16 items, with higher scores indicating greater movement confidence.

### Sample Size Calculations

We estimated that at 6 months following the structured interventions, at least a 30-minute increase per week would occur in the primary outcome of free-living MVPA (corresponding to an effect size of 0.32) for each of the tai ji quan interventions compared with stretching. This expected effect size (ie, a difference between groups of 30 min/wk of MVPA) is in line with prior estimates using self-reports^37^ and PA guidelines.^21,38^ With 80% power to detect 30 min/wk of MVPA at 12 months of follow-up (a 2-sided test with an α of .05, SD of 100, and nonsphericity correction of 0.8 for 4 repeated measures across 12 months), a total of 240 participants were required. Taking into account a 20% loss to follow-up at 12 months, we needed a total of 300 participants.

### Statistical Analysis

Participant demographic and health characteristics from baseline were summarized by intervention group with descriptive statistics (means with SDs and counts with percentages). Planned analyses involved estimating the tai ji quan intervention effect, relative to the stretching exercise control, on mean change in MVPA and secondary outcomes from baseline to follow-up at 12 months using the general linear mixed model with restricted maximum likelihood estimation. Repeated observations from participants were modeled using the compound symmetric correlation structure over time. Fixed effects included intervention group, time point (at baseline and at 4, 6, and 12 months), and group-by-time interaction. The intercept in each model was specified as random. The mixed modeling procedure allowed an intent-to-treat analysis that included all participants regardless of participation status, therefore producing unbiased estimates under an assumption of missing-at-random data. In a sensitivity analysis, missing data from all outcome measures were imputed using chained equations (eAppendix in Supplement 2).

Binary logistic regression was used to estimate the odds of meeting the recommended threshold of 150 min/wk of MVPA at 12 months between the tai ji quan intervention groups and the stretching group. Unadjusted estimates and their 95% CIs are presented for the primary outcome of MVPA and other study outcome measures. Because of the potential for type I error, findings related to the secondary outcomes presented in this study should be considered exploratory.

Three planned analyses and 1 ad hoc subgroup analysis examined whether the expected between-group differences in change in the primary outcome were associated with intervention compliance (defined by ≥75% of exercise class attendance), mental status (MMSE score ≤27),^39^ and mobility (Timed Up and Go [TUG] score ≥12 seconds)^40,41^ in a general linear mixed model with a 3-way group-by-subgroup-by-time interaction term. Using the same approach, the ad hoc analysis examined sex. *P* < .05 (2-sided) was considered significant. All analyses were performed with SPSS, version 29 (IBM Corp).

## Results

### Participant Characteristics

**Figure 1.**
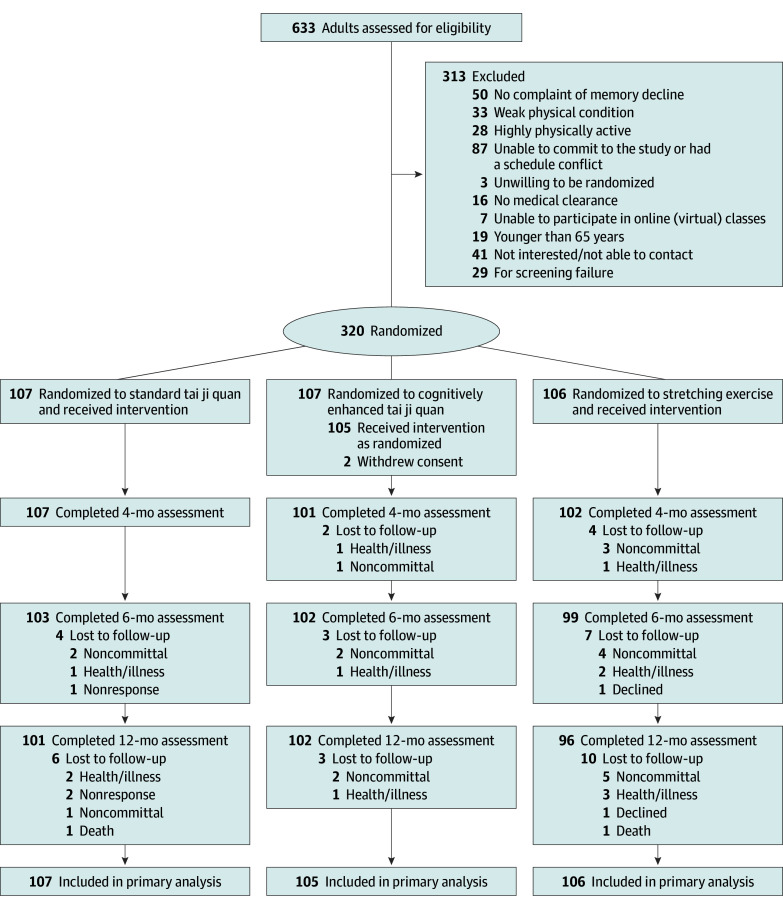
Participant Flow Diagram

Of the 633 individuals screened, 320 (50.6%) were eligible to participate and subsequently randomized to 1 of 3 intervention groups: 107 to standard tai ji quan, 107 to cognitively enhanced tai ji quan, and 106 to stretching (Figure 1). Two participants in the cognitively enhanced tai ji quan group withdrew consent and were excluded, resulting in 318 individuals enrolled. Baseline characteristics were similar among the groups (Table 1). Participants had a mean (SD) age of 76 (5) years; 212 (66.7%) were women and 106 (33.3%) were men. They identified as Asian (8 [2.5%]), Black (7 [2.2%]), Hispanic (14 [4.4%]), White (287 [90.3%]), or other race or ethnicity (16 [5.0%]).

**Table 1.** Baseline Characteristics of Randomized Participants by Intervention Group^a^

Characteristic	Intervention group (N = 318)		
	Standard tai ji quan (n = 107)	Cognitively enhanced tai ji quan (n = 105)	Stretching exercise (n = 106)
Age, mean (SD), y	75.9 (5.1)	76.0 (5.1)	76.0 (6.1)
Sex			
Female	66 (61.7)	75 (71.4)	71 (67.0)
Male	41 (38.3)	30 (28.6)	35 (33.0)
Race and ethnicity^b^			
Asian	3 (2.8)	1 (1.0)	4 (3.8)
Black or African American	1 (0.9)	2 (1.9)	4 (3.8)
Hispanic	6 (5.6)	4 (3.8)	4 (3.8)
White	95 (88.8)	100 (95.2)	92 (86.8)
Other^c^	8 (7.5)	2 (1.9)	6 (5.6)
Education			
High school diploma or less	38 (35.5)	41 (39.0)	39 (36.8)
Some college education	32 (29.9)	30 (28.6)	33 (31.1)
College degree or higher	37 (34.6)	34 (32.4)	34 (32.1)
BMI, mean (SD)	28.3 (5.0)	28.8 (6.0)	27.7 (5.6)
Resting blood pressure, mean (SD), mm Hg			
Systolic	127.3 (12.5)	126.1 (14.3)	126.1 (13.9)
Diastolic	74.0 (8.3)	73.5 (9.4)	73.8 (9.2)
Timed Up and Go score, mean (SD), s	12.7 (2.6)	12.6 (2.4)	12.5 (3.0)
GDS score, mean (SD)^d^	3.5 (2.9)	3.4 (2.9)	3.4 (3.3)
CDR score = 0.5^e^	82 (77)	82 (78)	83 (78)
MMSE score, mean (SD)^f^	27.2 (1.4)	27.3 (1.4)	27.2 (1.2)
No. of self-reported chronic conditions			
0	10 (9.3)	11 (10.5)	14 (13.2)
1	26 (24.3)	18 (17.1)	19 (17.9)
2	15 (14.0)	25 (23.8)	24 (22.6)
≥3	56 (52.3)	51 (48.6)	49 (46.2)
No. of self-reported medications used			
0	38 (35.5)	25 (23.8)	38 (35.8)
1	26 (24.3)	39 (37.1)	34 (32.1)
2	27 (25.2)	24 (22.9)	22 (20.8)
≥3	16 (15.0)	17 (16.2)	12 (11.3)
MVPA, mean (SD), min/wk	110.6 (88.9)	110.1 (85.7)	110.7 (86.6)

Abbreviations: BMI, body mass index (weight in kilograms divided by height in meters squared); CDR, Clinical Dementia Rating; GDS, Geriatric Depression Scale (Short Form); MMSE, Mini-Mental State Examination; MVPA, moderate to vigorous intensity physical activity.

^a^ Unless otherwise specified, values are presented as No. (%) of participants.

^b^ Values sum to more than 100% because participants could select more than 1 race or ethnicity.

^c^ Includes American Indian or Alaska Native or Native Hawaiian or Other Pacific Islander.

^d^ Consists of 15 items, with scores ranging from 0 to 15 (0-4, normal; 5-8, mild depression; 9-11, moderate depression; or 12-15, severe depression).

^e^ Measured on a scale from 0 to 3 points (0, none; 0.5, questionable; 1, mild; 2, moderate; or 3, severe).

^f^ Scores range from 0 to 30, with higher scores representing better global cognitive functioning.

A total of 118 participants (37.1%) reported having a high school diploma or less, and 162 (50.9%) reported having a gross family income of $39 999 or less. The mean (SD) MMSE score was 27 (1.3) points, and 247 (77.7%) participants had a CDR global score of 0.5. Approximately half of participants (156 [49.0%]) reported having 3 or more chronic conditions, and 118 (37.1%) were taking 4 or more medications. Participants had a mean (SD) TUG score of 12.6 (2.7) seconds for functional mobility, a mean (SD) ABC scale score of 82 (11.6), and a mean (SD) of 110.7 (86.6) min/wk of MVPA. Participants resided in urban (162 [51.0%]), suburban (70 [22.0%]), and rural (86 [27.0%]) communities across 81 cities and towns in 25 states across the US.

A total of 304 participants (95.6%) completed the trial. At 12 months of follow-up, data were available for 299 participants (94.0%). No intervention-related serious adverse events were noted (eAppendix in Supplement 2).

### Primary Outcome

**Figure 2.**
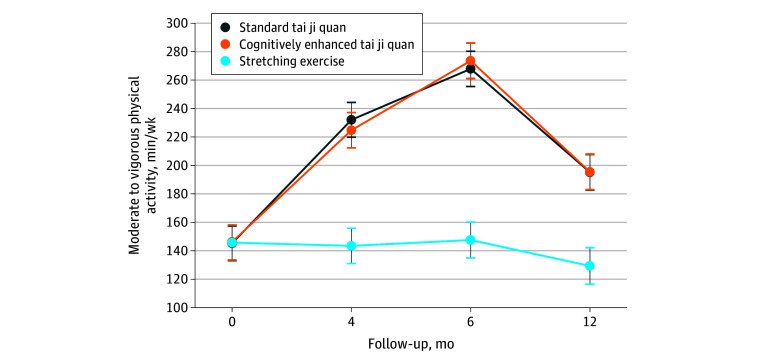
Changes in Engagement in Moderate to Vigorous Physical Activity Among Intervention Groups Across the Study Period

At 12 months of follow-up, the mean change in free-living MVPA from baseline was significantly higher in both the standard tai ji quan group (from 145 to 195 min/wk) and the cognitively enhanced tai ji quan group (from 146 to 196 min/wk) compared with the stretching control group (from 146 to 129 min/wk) (Figure 2). Mean differences of 66 min/wk (95% CI, 25-108 min/wk; *P* = .002) between the standard tai ji quan group and the stretching group and 65 min/wk (95% CI, 24-108 min/wk; *P* = .002) between the cognitively enhanced tai ji quan group and the stretching group were observed (Table 2). A nonsignificant decrease from baseline was observed for the stretching group (−16 [95% CI, −56 to 23] min/wk), and there was no significant difference in mean MVPA change between the 2 tai ji quan intervention groups (0.59 min/wk [95% CI, −42 to 41 min/wk]; *P* = .90). Similar patterns of mean change in MVPA from baseline to 6 months of follow-up (intervention termination) were observed (eTable 1 in Supplement 2). Both tai ji quan groups exhibited a significant decrease in mean MVPA from 6 to 12 months, with decreases of −72.7 min/wk (95% CI, −109.0 to −36.4 min/wk; *P* ≤ .001) with standard tai ji quan and −78.1 min/wk (95% CI, −114.3 to −41.8 min/wk; *P* ≤ .001) with cognitively enhanced tai ji quan (eTable 2 in Supplement 2).

**Table 2.** Primary and Secondary Outcomes at Baseline and Follow-Up and Between-Group Differences in Change From Baseline at 12 Months^a^

Measure	Intervention group, mean (SE) estimate			Between-group difference in mean change from baseline at 12 mo (95% CI)			
	Standard tai ji quan (n = 107)	Cognitively enhanced tai ji quan (n = 105)	Stretching exercise (n = 106)	Standard tai ji quan vs stretching exercise	*P* value	Cognitively enhanced tai ji quan vs stretching exercise	*P* value
MVPA, min/wk							
Baseline	145.4 (12.2)	146.3 (12.3)	145.8 (12.3)	NA	NA	NA	NA
Follow-up, mo							
4	232.1 (12.2)	224.8 (12.4)	143.5 (12.4)	NA	NA	NA	NA
6	267.9 (12.4)	273.6 (12.5)	147.6 (12.6)	NA	NA	NA	NA
12	195.2 (12.5)	195.5 (12.5)	129.4 (12.8)	66.2 (24.5-108.0)	.002	65.6 (23.8-107.5)	.002
Moderate PA, min/wk							
Baseline	110.6 (7.2)	109.4 (7.3)	110.9 (7.2)	NA	NA	NA	NA
Follow-up, mo							
4	145.5 (7.3)	142.2 (7.3)	103.1 (7.4)	NA	NA	NA	NA
6	163.1 (7.2)	163.1 (7.4)	105.6 (7.5)	NA	NA	NA	NA
12	131.9 (7.4)	135.0 (7.4)	100.9 (7.6)	31.4 (6.0-56.8)	.02	35.6 (10.2-61.1)	.006
Vigorous PA, min/wk							
Baseline	17.4 (3.9)	18.5 (3.9)	17.5 (3.9)	NA	NA	NA	NA
Follow-up, mo							
4	42.3 (3.9)	41.3 (4.0)	20.2 (4.0)	NA	NA	NA	NA
6	52.5 (3.9)	55.3 (4.0)	21.1 (4.0)	NA	NA	NA	NA
12	31.6 (4.0)	30.3 (4.0)	14.4 (4.1)	17.2 (3.3-31.2)	.01	14.9 (1.0-28.8)	.03
Movement confidence							
Baseline	82.6 (1.3)	82.4 (1.3)	81.6 (1.3)	NA	NA	NA	NA
Follow-up, mo							
4	86.6 (1.3)	85.0 (1.3)	82.8 (1.3)	NA	NA	NA	NA
6	88.3 (1.3)	89.5 (1.3)	83.1 (1.3)	NA	NA	NA	NA
12	88.4 (1.3)	89.9 (1.3)	82.6 (1.3)	4.9 (1.6-8.2)	.004	6.5 (3.2-9.8)	<.001

Abbreviations: MVPA, moderate to vigorous physical activity; NA, not applicable; PA, physical activity.

^a^ Intention-to-treat analysis using mixed models.

### Secondary Outcomes

At 12 months of follow-up, both tai ji quan groups had a significantly greater level of change in moderate intensity activity compared with the stretching group, with mean differences in change from baseline of 31 min/wk (95% CI, 6-57 min/wk; *P* = .02) between the standard tai ji quan group and the stretching group and 36 min/wk (95% CI, 10-61 min/wk; *P* = .006) between the cognitively enhanced tai ji quan group and the stretching group. Both tai ji quan groups also had a significantly greater level of change in vigorous intensity activity compared with the stretching group, with mean differences in change from baseline of 17 min/wk (95% CI, 3-31 min/wk; *P* = .01) for the standard tai ji quan group and 15 min/wk (95% CI, 1-29 min/wk; *P* = .03) for the cognitively enhanced tai ji quan group. For change in self-reported movement confidence, both tai ji quan groups scored significantly higher than the stretching group, with mean differences in change in ABC from baseline of 5 points (95% CI, 2-8 points; *P* = .004) between the standard tai ji quan group and the stretching group and 7 points (95% CI, 3-10 points; *P* < .001) between the cognitively enhanced tai ji quan group and the stretching group. Full results are presented in Table 2.

At 12 months of follow-up, 72 of 107 participants (67.3%) in the standard tai ji quan group, 75 of 105 (71.4%) in the cognitively enhanced tai ji quan group, and 42 of 106 (39.6%) in the stretching group met the MVPA guideline. Compared with the stretching control group, odds ratios for meeting the MVPA guideline were 3.11 (95% CI, 1.75-5.53; *P* < .001) for the standard tai ji quan group and 3.67 (95% CI, 2.02-6.65; *P* < .001) for the cognitively enhanced tai ji quan group.

### Additional Analysis

In sensitivity analysis, point estimates derived from the multiple imputation method were similar to those generated via the mixed modeling approach (eTable 3 in Supplement 2). Subgroup analyses of modifiers of intervention effects on the primary outcome were conducted. The intervention effects of tai ji quan did not differ by intervention compliance, mental status, mobility, or sex (eTable 4 in Supplement 2).

## Discussion

In this study of community-dwelling older adults with MCI or self-reported memory concerns, 6 months of either a standard or cognitively enhanced tai ji quan intervention, compared with stretching, led to a mean increase of 65 min/wk from baseline in free-living MVPA observed 6 months post intervention. In an exploratory analysis, we also observed increases in dose-specific PA (ie, moderate intensity, vigorous intensity) among tai ji quan participants compared with stretching participants at 12 months. Participants who received either type of tai ji quan training, relative to those receiving the stretching control, were 3 times more likely to have achieved the recommended amount (≥150 min/wk) of MVPA at 12 months. Subgroup analyses indicated that the effect of the tai ji quan intervention on increasing MVPA was not mediated by exercise compliance or moderated by participants’ level of global cognitive function, mobility, or sex differences.

Findings from this study corroborate prior intervention and dissemination studies in which participants in either tai ji quan or PA interventions showed continued tai ji quan or other PA behaviors after termination of structured interventions.^22,23,24^ Other studies have either reported no continued PA behavior or maintenance^42^ or a substantial decreasing trend in PA at follow-up.^43^ A scarce number of clinical investigations exist that target older adults with memory concerns, such as MCI, for whom physical inactivity is more prevalent^9,10,11,12^ but often not being targeted for PA intervention. To our knowledge, our study is one of the few that address this research gap. Our findings provide support for PA promotion in that tai ji quan, which offers many other therapeutic benefits to older adults,^19,44^ can be implemented to promote free-living MVPA among older adults with MCI or memory concerns in home settings.

Our PA measure, assessed with the IPAQ-S, does not contain tai ji quan activity. The underlying mechanisms through which the tai ji quan intervention served as a gateway to improved MVPA following the intervention period are currently an open question. One hypothesis is that tai ji quan training improved participants’ movement confidence, as shown by ABC scores, and induced them to become more physically active. Prior research supports that exposure to an exercise intervention is a source of efficacy information or motivation^45^ and, subsequently, helps boost exercise participation^46,47^ and encourage maintenance of long-term PA behavior.^47,48^ Alternatively, evidence has pointed to the potential role of improved function, including dual-task ability (eg, during walking)^20,49^ or mobility,^27^ in increasing older adults’ motivation to try other forms of activity. Both may result from the physical and cognitive responses to concurrent motor control and attention demands that are inherent features of tai ji quan training.^50^

The finding that tai ji quan enhances MVPA among older adults with MCI or memory concerns following structured interventions at a level beyond current PA guidelines is of high public health importance. Participation in regular PA is associated with lower risk of all-cause and cause-specific mortality.^51^ PA is also effective in improving physical and cognitive functioning^2^ and in reducing cognitive decline and risk for developing dementia.^17,18,52,53^ For older adults with MCI or memory concerns, the health benefits of increases in free-living PA can help them live independently; maintain their levels of daily functioning, including balance and coordination; and prevent or manage chronic disease.^21,54^ In light of the limited evidence-based programs to improve PA for older adults with memory problems, this study suggests that a home-based, twice-weekly tai ji quan intervention, delivered virtually, may offer a promising approach for increasing PA levels in the short term. Therefore, tai ji quan may be prescribed by clinicians as a means of promoting free-living PA in the community and managing MCI or memory concerns in clinical practice.^55,56^

### Strengths and Limitations

This study has several strengths. First, this is one of the few trials to use a remote approach to intervention delivery, evaluation, and study outcome assessments. Second, participants were recruited from geographic regions throughout the US, which increases generalizability. Third, the resulting increase in PA suggests that a home-based intervention can be used not only to improve cognitive function^20^ but also to promote sustained behavior change in older adults with MCI, a population characterized by physical inactivity.^9,10,11,12^

The study also has limitations. First, the MVPA measure was self-reported, a method that is known to be subject to recall bias and overestimation.^57^ Second, tai ji quan is considered moderate intensity PA and we did not explicitly exclude individuals who engaged in this level of activity. Third, follow-up was limited to 6 months post intervention. Whether the increased MVPA time shown contributes to long-term MVPA behavior change remains to be determined. Next, because the study was conducted during the COVID-19 pandemic, the activities recorded during the trial period may not represent a full range of PA that would be captured during nonpandemic times. Finally, the study sample comprised mostly White, female, and educated individuals, which may limit the generalizability of the findings presented.

## Conclusions

In this secondary analysis of a randomized clinical trial, a structured tai ji quan intervention delivered virtually in a home setting to older adults with MCI or self-reported memory concerns increased their MVPA behavior at 6 months following cessation of the intervention. These findings suggest that tai ji quan may be promoted as an avenue to achieve PA guidelines for older adults with MCI or subjective memory concerns.

## Notes

Supplement 1.
Trial Protocol

Supplement 2. **eTable 1.** Estimated Means on Primary and Secondary Outcomes at Baseline, 4-Month, 6-Month, and 12-Month Follow-Ups and Between-Group Differences in Change From Baseline at 12 Months (Intention-to-Treat Analysis Using Mixed Models)—With Time-Specific Effects **eTable 2.** Estimated Means on Moderate to Vigorous Intensity Physical Activity at 6-Month and 12-Month Follow-Ups and Within-Group Differences in Change From 6 Months to 12 Months (Intention-to-Treat Analysis Using Mixed Models) **eTable 3.** Estimated Means on Primary and Secondary Outcomes at Baseline, 4-Month, 6-Month, and 12-Month Follow-Ups and Between-Group Differences in Change From Baseline at 12 Months (Intention-to-Treat Analysis Using Multiple Imputation) **eTable 4.** Statistical Test Results From the Planned Subgroup Analyses Involving Group by Subgroup (Intervention Compliance, Mobility, Mental Status, and Sex) by Time on Moderate to Vigorous Intensity Physical Activity **eAppendix.** Psychometrics, Adverse Events, and Data Imputation

Supplement 3.
Data Sharing Statement
