# Supplementary material for: Physical Activity Engagement After Tai Ji Quan Intervention Among Older Adults With Mild Cognitive Impairment or Memory Concerns: A Secondary Analysis of a Randomized Clinical Trial
Source: JAMA Netw Open. 2024 Dec 17;7(12):e2450457. doi: 10.1001/jamanetworkopen.2024.50457 (PMC11653122; doi:10.1001/jamanetworkopen.2024.50457)
Supplement: Supplement 1. — Trial Protocol [file jamanetwopen-e2450457-s001.pdf]

Supplement 1

Trial Protocol

Dated: October 13, 2024

Li F, Harmer P, Eckstrom E, Winters-Stone K. Moderate to vigorous physical activity in older adults with mild cognitive impairment and self-reported memory concerns: a secondary analysis of a randomized clinical trial

This study protocol, both original and modified (due to COVID-19), has been assembled to provide readers with additional information about the trial design, methodologies, and statistical analyses relevant to both our primary outcome study and the current study reported in this article.

Original Trial Protocol

Protocol title

Cognitively enhanced tai ji quan training on global cognitive function and lower dual-task costs during walking in older adults with mild cognitive impairment: A randomized clinical trial

Study design

The design is a prospective, assessor-blinded, 3-arm parallel group, randomized, pragmatic trial with a 1:1:1 allocation ratio.

Condition of study

Older adults with mild cognitive impairment (MCI)

Trial Funding

This trial is funded by the National Institute on Aging (R01AG059546), National Institutes of Health.

Trial registration

ClinicalTrials.gov number: NCT04070703: Released August 28, 2019

Trial protocol review and approval

The original in-person trial protocol, developed in 2018, was fully reviewed by the Institute Review Board (IRB) of Oregon Research Institute (ORI). The protocol received an annual IRB review during each year of the project. Mandated by the National Institute on Aging (NIA), a Safety Officer, appointed and approved by the NIA, also reviewed the study protocol, oversaw the safety and scientific integrity of the study, and received annual updates on the progress made on the various aspects of the project activity. The protocol was approved on January 23, 2019, by the IRB of ORI (IRB Registration No. 00000278) and subsequently approved by the NIA-appointed Data Safety Monitoring Officer. Details of the trial protocol have been published.[1]

Primary study objective

To examine whether participation in a 6-month virtual tai ji quan-based intervention, compared with a stretching exercise control, would result in increases in the level of moderate-vigorous intensity PA (MVPA) during post-intervention follow-up following the termination of the structured intervention in older adults with MCI.

Secondary study objective

To examine the proportion of tai ji quan participants who meet the recommended level of MVPA post-intervention follow-up.

Study Population and eligibility criteria

Population: community-dwelling older adults with MCI.

Inclusion criteria: (a) being 65 years or older; (b) having a reported memory decline (by the participant and/or an informant); (c) having a diagnosis of MCI by a global score of ≤0.5 on the Clinical Dementia Rating (range: 0-3);[2] (d) showing no diagnosis of dementia or significant cognitive impairment, as indicated by a score of ≥24 on the Mini Mental State Evaluation (MMSE, range: 0-30).[3]

Exclusion criteria: (a) having no medical clearance from a healthcare provider; (b) having participated in structured (class-based, instructor-led) rigorous activities or muscle-strengthening activities (>2 times a week for >15 minutes per session) in the past 3 months; (c) showing clinically

significant depression (>4 on the Geriatric Depression Scale; range: 0-15);[4] (d) having any physical condition that would preclude participation in moderate-intensity exercise; and (e) being unwilling to commit to the duration of the intervention or accept group assignment.

Recruitment sources and procedures

Recruitment strategies to identify potential study participants include promotions at local senior/community centers, meal sites, offices of healthcare professionals and medical clinics, targeted mass mailings, organizational websites, and local newspaper advertisements. To reduce potential expectation bias, participants will be informed that the study compares three different exercises and that they will be assigned to one of these exercise groups at random.

To ascertain eligibility, a research assistant will make an initial telephone contact with those who respond to the study promotion. This initial prescreen telephone contact will determine basic eligibility set forth under the recruitment inclusion and exclusion criteria. Potentially, individuals who meet initial inclusion criteria will be scheduled for a 2-hour in-office visit at our research office, where the remainder of the screening work (i.e., CDR, MMSE) will be completed, along with baseline assessment if the participant is qualified.

Randomization, allocation, concealment, and blinding

Eligible participants will be randomized to 1 of the 3 intervention groups with an allocation of 1:1:1 through a permuted block randomization with a block size of 3 or 6 to prevent anticipation of assignment to the study condition. Concealment of allocation will be implemented. The project data analyst will use computer software (nQuery) to generate a randomization schedule, which will be kept in a sealed envelope. On the day of the assignment, a research assistant will assign qualified individuals to intervention groups. The allocation sequence assigns individuals in the order that they were scheduled for baseline assessment. Randomization will occur after informed consent is obtained and baseline assessments have been completed.

Project data analysts will be blinded to group allocation. All study assessors who collect study outcome measures will also be blinded to the main study design and group allocation. Blinding will be strictly maintained by emphasizing to assessors the importance of minimizing assessment bias and regular checking of the blinding status. Efforts will be also made to maintain separation between the study assessors and research assistants who deal with administrative activities and class safety monitoring, and between study assessors and class instructors who deliver the intervention classes. Because of the nature of the interventions, study participants and interventionists will not be masked to group allocation. Participants will be instructed not to reveal their group status to the assessment staff at any time during follow-up.

Intervention

Standard tai ji quan

This conventional tai ji quan intervention is conducted twice weekly, for 60 minutes per session for a total of 24 weeks (6 months). Training exercises include practice of tai ji quan forms with synchronized breathing, supplemented by a set of mini-therapeutic exercises.[5] Specifically, the training involves repeated practice of (a) symmetrical, coordinated, trunk-driven tai ji quan form movements, (b) controlled displacement (weight-shifting) of the body’s center of mass over varying sizes of the base of support, (c) dynamic eye-hand movements during whole-body motion, and (d) multidirectional (anterior-posterior and medial-lateral) stepping. As a balance-based training program, movement practices emphasize a dynamic interplay of stabilizing and self-induced destabilizing postural actions and balance exercises that target mobility, stability limits, and sensory integration.

Cognitively enhanced tai ji quan

With the same exercise frequency and duration as in standard tai ji quan, this intervention explicitly integrates cognitive training exercises.[1] Specifically, tai ji quan training exercises include

repeated practice of (a) symmetrical postural forms/movements synchronized with breathing, (b) controlled displacement (weight-shifting) of the body's center of mass over the base of support, (c) dynamic eye-hand movements during whole-body motion, (d) multidirectional (anterior-posterior and medial-lateral) stepping, and (e) rotational ankle sway and self-induced reactive postural recovery actions. These dynamic exercises are superimposed, concomitantly, with a mix of interactive, cognitively stimulating, dual-task practices that challenge attention control, working memory, verbalization, response inhibition, processing speed, dual tasking, task switching/prioritization, and spatial orientation and postural awareness.

Stretching exercise

The stretching exercise intervention consists of breathing, stretching, and relaxation.[1,5] The core exercises encompass a variety of light and static stretches for joints and muscles, performed in a seated or standing position, that involve the upper body (arms, neck, upper back, shoulder, back, and chest), lower extremities (quadriceps, hamstrings/calfs, and hips), and gentle and slow trunk rotations. Also included are intermittent light walking, deep abdominal breathing exercises that emphasize inhaling and exhaling to maximum capacity, and progressive relaxation of major muscle groups.

Duration of intervention

The entire duration of the intervention lasts 6 months

Follow-up schedule

Follow-ups are scheduled as follows:

- 4 months (mid point)
- 6 months (intervention termination) and
- 12 months (post-intervention follow-up)

Primary outcome

The study has the following the primary outcome:

MVPA assessed using the International Physical Activity Questionnaire (IPAQ) – short form.[6] Participants are asked to report how often (measured in days) and how much time (in munities) over the previous week in which they participate in physical activity or exercise of any moderate (gardening, cleaning, bicycling at a regular pace, swimming or other fitness activities) or vigorous (heavy lifting, heavier garden or construction work, chopping woods, aerobics, jogging/running or fast bicycling) intensity that lasted at least 10 minutes at a time. The activities to be asked during the post-intervention follow-up (at 12 months) will not include the time spent in attending the assigned exercise programs in the study. Total weekly duration of MVPA, measured in minutes, are calculated by multiplying frequency and duration (vigorous activity weighted by 2) to form a total weighted amount of MVPA min/week.

Secondary outcomes

The study has the following the secondary outcomes:

1. moderate-intensity PA (min/week),
2. vigorous PA (min/week),
3. meeting 150 min/week MVPA guidelines (≥150 min/week), and
4. movement confidence measured by the Activities-specific Balance Confidence (ABC) scale[7]

The ABC assesses one's confidence in performing various daily activities (e.g., picking up an object from the floor, standing on a chair to reach for something, walking on icy sidewalks) without compromising one's balance. The scale, which has established psychometric properties,[7] is rated by participants on a scale of 1 (not at all confident) to 10 (completely confident) and the resulting scores are computed as the average level of confidence participants have across 16 activities with higher scores indicating greater movement confidence.

Covariates

There are no pre-specified covariates in this study.

Assessment of intervention adherence

Intervention classes will be closely monitored by research staff throughout the study period. Session-by-session attendance of the study participants will be recorded by research staff, regardless of participants' participation status, and this information on exercise adherence rate will be used as an indicator of intervention adherence across the three intervention groups. For each intervention group, there will be a total of 48 exercise classes to be completed over the 24-week intervention period. Accordingly, the average intervention adherence rate will be calculated as follows: number of intervention sessions attended during the 24 weeks divided by total number of sessions prescribed for the study, reported as a percentage.

Baseline measures

At baseline, demographic and other information about age, sex, ethnicity/race, income, education, living arrangements, medical conditions, health status, habitual physical activity,[6] movement confident,[7] and residence location will be collected via a survey questionnaire. Body weight, assessed by a medical scale, and height, measured by with a ruler fixed on a wall, will also be ascertained. These data will be used to describe study population characteristics at baseline.

Statistical analysis

Participants' demographic and health characteristics assessed at baseline will be summarized by intervention group with descriptive statistics (means, standard deviations, counts, or percentages). Preplanned analyses will involve estimating the intervention effect of tai ji quan interventions on change in MVPA from baseline to follow-up at 12 months using the general linear model with repeated measures (baseline, 4 months, 6 months, and 12 months). Planned comparisons of MVPA and secondary outcomes of moderate, vigorous PA and movement confidence on change at 12 months from baseline between each of the tai ji quan interventions relative to stretching control are made. Binary logistic regression is used to estimate the odds of meeting the recommended threshold of 150 min/week of MVPA at 12 months between tai ji quan interventions and stretching groups using the imputed datasets. Unadjusted estimates and their 95% confidence intervals (95% CIs) will be presented for the primary outcome of MVPA and other study outcome measures.

Sub-group analysis. Three planned subgroup analyses will be performed to examine whether the expected between-group differences in change in the primary outcome was associated with intervention compliance (≥75% of exercise class attendance), mental status (MMSE) ≤24), and mobility (Timed Up&Go (TUG) ≥12 seconds)[8] in a general linear mixed model with a three-way interaction term of Group by Subgroup by Time. No Bonferroni adjustments will be made in all null hypothesis testing analyses. All statistical tests will be 2-sided with a $P < .05$ significance level. Assuming data to be missing at random, outcome variables will be analyzed with intention-to-treat with missing data imputed (ten imputed datasets) using chained equations. All data analyses are to be performed with SPSS version 26 (IBM Corp., Armonk, NY, USA).

Power. The target sample size was calculated for the main outcomes of the 6-month tai ji quan trial,[1] with 80% power for a medium effect size (Cohen $d = 0.5$) and an expected loss to follow-up rate of 15%. Although no a priori sample size calculations for this follow-up secondary analysis study were conducted, post hoc, we estimated that, at 6 months following the structured interventions, at least a 30-minute increase per week in free-living MVPA (corresponding to an effect size of 0.32) for each of the tai ji quan interventions compared with stretching exercise. This expected effect size is in line with prior estimates using self-reports[9] and recommended levels of physical activity.[10] With 80% power to detect a 30 min/week MVPA (a 2-sided test with an alpha of 0.05, standard deviation of 100, a non-sphericity

correction of 0.8 for 4 repeated measures across 12 months), a total of 240 participants were required. Taking into account a 20% loss to follow-up at 12 months, we need a total of 300 participants.

Treatment of noncompliance and missing data

All enrolled study participants will be followed until the trial period ends, whether or not the participant is still receiving or complying with the intervention. Primary endpoint analyses will be conducted according to the original randomization scheme in an intention-to-treat approach. We will use a multiple imputation data method for missing data. We plan to perform 10 sets of imputations. Variables without missing data to be used for prediction of imputed values on the study variables will include baseline measures of age, sex, level of education, MMSE, number of chronic conditions, and depression. The imputed data will be submitted to the same analytic model (i.e., general linear model) as the non-imputed observed data, and the results of each analytic model will be pooled across the 10 imputed data sets. The secondary outcome analyses will also be conducted using the multiple imputation method described above. Tertiary outcome analysis will be conducted with observed data without imputation.

Statistical software

Statistical analyses will be performed with the use of Stata software (version 17, Stata Corp), SPSS software (version 25, SPSS), and Mplus software (version 8.4, Muthén & Muthén).

Activities during post-intervention follow-ups

No physical activities are prescribed to the study participants during post-intervention follow-up at 12 months.

Adverse events monitoring

Throughout the study period, both intervention- and non-intervention-related adverse events will be closely monitored and recorded by research staff and adjudicated by the Principal Investigator of the study. We will classify adverse events in three categories: Mild, Moderate, and Serious. Similarly, for all events observed or reported in this study, we will further classify them into three categories in relation to the intervention: Unrelated, Possible, or Definite. The following section describes the classification and categorization of our study adverse events. Per our IRB protocol, any serious adverse events will be reported to the IRB and the project Safety Officer within 48 hours of the reported incident.

Adverse Event and Serious Adverse Event Collection Process

The project defines Adverse Events and Serious Adverse Events as follows:
Definition of Adverse Events may include the following:

- Any musculoskeletal pain or discomfort, including lower back pain, ankle/muscle soreness or pain, or a fall without needing medical attention
- Any event that requires medical treatment but is not an immediate life-threatening condition (e.g., eye surgery or a medical procedure)

Definition of Serious Adverse Events are undesirable experiences associated with the prescribed exercise interventions in this project. Serious Adverse Events may include the following:

- Death
- Falls that result in a serious injury that requires immediate medical attention
- Prolonged hospitalization
- Important medical or life-threatening events such as a heart attack that require treatment in an emergency room
- Events that cause persistent or significant disability or incapacity

Classification of Adverse Events

This project will classify any adverse events observed during the 6-month active exercise intervention period into the following three categories:

- Mild: events that require no medical treatment or are non-life threatening
- Moderate: events that require medical treatment but are not immediate life-threatening conditions
- Serious: events that result in death or are life threatening and require medical treatment, including prolonged hospitalization or significant disability/incapacity

Relatedness

For all events observed or reported, the project will further classify them into three categories in relation to the intervention:

- Not related: an event that is reported but not directly related to participation in the intervention
- Possibly related: an event that is observed during an exercise class that is considered likely to be associated with participation
- Definitely related: an event that is observed or reported during an exercise class and is considered directly related to participation.

Adverse Events and Serious Adverse Event (SAE) Reporting Process

We will carefully monitor for unexpected adverse events, as well as the expected outcomes, that participants experience during each online exercise session and outcome assessment, whether or not the events or outcomes are directly related to the study intervention. All symptoms reported by the study participants will be tabulated in a checklist format and recorded by a designated research assistant. When an adverse event is identified, the staff member will report it directly to the Principal Investigator (PI). An Adverse Event Report Form will immediately be completed, filed, and reported to PI.

The PI will be directly responsible for monitoring and documentation of adverse events during the implementation of all prescribed exercise sessions across the three study conditions. Once the project is implemented, any activities (during a class session or during an assessment session) resulting in participant distress or discomfort, exercise-related side effects, injuries, or falls will be reported immediately to the PI, who will, upon consultation with other members of the team, take appropriate action. This will include consultation with the team's medical expert. The PI will report such incidents immediately to the IRB Chair at ORI. Summary reports of adverse events, and any subsequent IRB action taken as a result of such events, will be routinely provided to the NIA PO and the NIA-appointed Safety Officer.

For each event observed or reported, regardless of setting where the event takes place (i.e., at home or during exercise classes), a designated research assistant will contact the participant within 3 working days, via a telephone call, to ascertain detailed information related to the event. During this phone contact, information about date/time, location, nature of the event, symptoms experienced, and any measures taken for the event will be collected and documented. If necessary, the informant for the study participant will be contacted to verify the information provided by the participant. The recorded event, along with a detailed description, will be tabulated in a checklist format and entered into the project's Adverse Event Log. It will be shared immediately with the PI, who will report, within 3 working days, to the IRB, NIA-appointed Safety Officer, and Project Officer at NIA.

The following summarizes the reporting procedure to be implemented in this project:

- Expedited reporting: All SAEs (including expected and related or possibly related) observed during the active study period will be reported to local IRB and Safety Officer within 3 working days.
- Routine reporting: All events will be included in routine reports.

Fidelity of intervention delivery

A standardized intervention protocol and a process evaluation checklist, developed via prior trials,[5] will be implemented. These measures focus primarily on intervention fidelity and involve issues such as (a) interventionist qualifications and training, (b) teaching quality of the individual forms/movements or routine in each session, (c) exercise intensity and consistency in training dosage across different sites, and (d) weekly class attendance checking and monitoring. The evaluation will be conducted monthly by either an authorized research team member or an instructor, per guidelines specified in an established fidelity checklist.[10] In evaluating the item in (b), high intervention fidelity will be considered achieved if at least 95% of the mandatory components (overall completion of pre-specified activities, quality of verbal and visual instructions, emphasis of core training points, session completion time) are fully or partially delivered in each session.

Attention control across intervention groups

Participants assigned to each of the three intervention groups will receive the same amount of contact time from class interventionists and research staff. Specifically, 48 exercise sessions are planned for each exercise program in the study. Assessment time at each assessment time point will be constant (i.e., around 2.5 hours per visit). Unless there are special circumstances (e.g., participants experience an adverse event or illness), there will be no additional contact time with the project instructors or staff.

Trial interventionists

The community exercise instructors who deliver all three exercise interventions will be trained per our previously established criteria.[5] These trial interventionists will be trained by the Principal Investigator initially via an 8-hour training and orientation workshop, during which detailed instructions about the specifics of program delivery, practice safety, and teaching requirements will be provided. In-service training will be provided on a monthly basis and when needed as determined by the investigators. These trial interventionists will also be asked to maintain the confidentiality of the study participants in their classes.

Trial outcome assessors

All research outcome assessors will complete a computer-based training course through Oregon Research Institute, an educational requirement for all researchers who conduct or support research involving human subjects. This will be completed during the project start-up period before data collection. In addition, these assessors will complete an online training on the clinical administration of cognitive-based measures (i.e., CDR, Montreal Cognitive Assessment (MoCA)[11] and receive training from the Principal Investigator on established assessment procedures related to all study outcome measures including self-reports and performance-based measures.

Intervention adherence and attrition

The overall intervention adherence (i.e., exercise class attendance) rate is defined as the sum of the total number of participants attending divided by the maximum number of 48 sessions planned, multiplied by 100, during the 6 months of active intervention. For the trial, we will strive for an adherence rate of ≥75%. Accordingly, class attendance across the three study conditions will be closely monitored on a weekly basis. As an adherence procedure, participants who miss two consecutive sessions will be contacted by phone to ascertain the reason(s) for their absences and to encourage them to return.

Unavoidable drop-outs, from causes such as death, onset of severe illness, changes in health conditions, or other medical complications, are anticipated during the course of our active intervention. Based on our prior trial records,[10] we estimate a 15% intervention dropout rate for the overall study (i.e., 15% of the total enrolled participants will withdraw from or stop attending assigned exercise classes).

Study retention

Study retention is defined as participants who voluntarily provide primary outcome data regardless of intervention participation status. For the trial study, we have planned an overall study retention rate of 85%. To accomplish this goal, planned measures will be taken to ensure that as many participants as possible, including dropouts, attend each scheduled in-person assessment visit at our research facilities. Our proactive methods will include frequent telephone and e-mail contacts with subjects who miss scheduled data assessment appointments.

Protocol adherence

The overall intervention adherence (i.e., exercise class attendance) rate is defined as the sum of the total number of participants attending divided by the maximum number of 48 sessions planned, multiplied by 100, during the 6 months of active intervention. For the trial, the target adherence rate is ≥75%. Class attendance across the three study conditions will be closely monitored on a weekly basis by research staff and reviewed on a monthly basis by key personnel of the project. Participants who miss two consecutive sessions will be contacted by phone to ascertain the reason(s) for their absences and to encourage them to return.

Intervention fidelity

Intervention fidelity will focus primarily on issues such as (a) interventionist qualifications and training, (b) teaching quality of the individual forms/movements or routine in each session, (c) exercise intensity and consistency in training dosage across different sites, and (d) weekly class attendance checking and monitoring. The evaluation will be conducted monthly by either an authorized research team member or an instructor, per guidelines specified in an established fidelity checklist.[5] In evaluating the item in (b), high intervention fidelity will be considered achieved if at least 95% of the mandatory components (overall completion of pre-specified activities, quality of verbal and visual instructions, emphasis of core training points, session completion time) are fully or partially delivered in each session. Deviations related to intervention delivery protocol will be captured on a protocol deviation form and be entered into a project database for evaluation by the research staff and Principal Investigator. Prompt action will be taken by the Principal Investigator to remedy any problems and deviations identified.

Data completeness and quality control

Data completion refers to the study measurement ascertainment status from all enrolled study participants. The project strives to collect study outcome measures on every participant at each scheduled time point (baseline, 4 months, 6 months, and 12 months), regardless of his/her intervention participation status. As specified in the protocol for the study, we have planned an overall study retention rate of 85% (i.e., having primary and secondary outcome measures available on 85% of the enrolled participants and their informants). To accomplish this goal, planned measures will be taken to ensure that as many participants as possible, including dropouts, attend each scheduled in-person assessment visit at our research facilities. Our proactive methods will include frequent telephone and e-mail contacts with subjects who miss scheduled data assessment appointments.

Outcome measures ascertained will be closely checked and verified for accuracy and completeness via a rigorous data checking and reviewing process implemented by the trial analyst(s) who will be blinded to group allocation. Specifically, data quality and accuracy will be assured via the following steps.

- All assessment data (i.e., surveys, semi-interviews, observational assessments) will be double checked for errors (e.g., out-of-range data, missing data, and accuracy) by research assistants before they are entered into a database.
- Raw data will be entered twice via an existing data entry module.

- All entered data will be subject to a process that checks for accuracy and consistency and will be verified by the project statistician. In the event that data entry errors are discovered, additional charts will be randomly selected for internal review.
- Raw data entry, cleaning, coding, manipulation, verification, and merging from all four time points (baseline, 4 months, 6 months, and 12 months) of assessment will be completed within 5 days after each follow-up.
- Charts and plots will be presented to the study's statistician and Principal Investigator every three months for quality assurance.

In addition, deviations related to outcome assessment and data ascertainment protocols will be captured on a protocol deviation form and be entered into a project database for review and evaluation by the research team and Principal Investigator during quality assurance meetings, which will be held on either a monthly or quarterly basis. Prompt action will be taken by the Principal Investigator and team's statistician to remedy any problems and deviations identified, and they will perform follow-up evaluations of actions taken, if necessary.

Intervention safety and analysis

Throughout the study period, both intervention- and non-intervention-related adverse events will be closely monitored and recorded by research staff and adjudicated by the Principal Investigator. We will classify adverse events in three categories: Mild (i.e., events that require no medical treatment or are not life threatening), Moderate (i.e., events that require medical treatment but are not immediate life-threatening conditions), and Serious (i.e., events that result in death or are life threatening and require medical treatment, including hospitalization, or significant disability/incapacity). For all events observed or reported, we will further classify them into three categories in relation to the intervention: Unrelated (an event that is reported but not directly related to participation in the intervention), Possible (an event that is observed during a class and that is considered likely to be associated with participation), or Definite (an event that is observed or reported during a class and is considered directly related to participation).

Per our IRB protocol, any serious adverse events that we have collected during the entire course of intervention will be reported to the IRB and the project Safety Officer within 48 hours of the reported incident. This will also include unanticipated issues such as prolonged hospitalizations and deaths. Safety analysis will involve tabulating the occurrence of adverse events, including Serious adverse events (deaths and hospitalizations) and unanticipated problems/issues among the three groups. Given the low risk of the trial, no inferential statistical tests are planned for safety.

Data Management

All data collected (self-reports, interviews, recorded study outcome performance ratings or scores) from the project will first be stored in locked filing cabinets in a designated area inside the ORI office building and, after review for completeness and accuracy, will be entered into a secure, password-protected ORI computer network database to be established by the data team staff. Only authorized project staff conducting this project will have full access to the data collected. Participants and informants' responses to the project surveys, interviews, and other forms of data will be coded with a unique numeric identification code. Only designated project staff will have the key to the file cabinets or password to the database and will have access to the data only for status checking or data verification purposes. An Excel data system will be created to track the subjects' status related to recruitment, enrollment, intervention participation, and follow-up assessment. For all raw data, a system file will be created, using the SPSS statistical software, that merges various subsets of data for final analyses. A periodic security check on the data files will be conducted under the supervision of project's statistician. Only the designated data analyst, project biostatistician, and PI will have access to the raw data and analyses.

Adverse Events Reporting

The following summarizes the procedures that will be followed by the investigative team when a participant experiences an adverse event (during the entire course of the study project):

1. The participant will be encouraged to call ORI's research staff.
2. ORI's staff will file an incident report and immediately report the event to the Principal Investigator.
3. The Principal Investigator will inform ORI's IRB administrator and project Safety Officer appointed by NIA of the incident within 48 hours.
4. The incident report will be sent to the Project Officer at NIA and filed in the project database.
5. An ORI staff member will conduct a follow-up contact with the participant within 5 working days to reassess the situation and report back to the Principal Investigator.

Protocol Modifications

The original trial involved an in-person protocol. That is, all research activities related to recruitment, enrollment, intervention, intervention safety monitoring, and outcome assessments were planned to be conducted with an in-person method at the ORI facilities. However, due to the COVID-19 outbreak modifications were made to the original trial protocol.[1] Detailed description is provided below. where appropriate, modifications relevant to the current study are indicated at each section.

Background

On March 15, 2020, due to the widespread nature of the COVID-19 pandemic and for the safety and wellbeing of trial participants and project staff, the in-person study trial protocol (see above), approved by ORI's IRB and with notification given to the funding agency, was completely suspended. At the time of the suspension, we had a total of 28 participants who were 3 months into the active intervention.

Upon IRB approval, on March 30, 2020, all research activities were resumed by moving the trial activities online, delivered at home through Zoom videoconferencing. The decision to switch from in-person to online delivery was made by the investigative team based on (a) the need to adapt to the COVID-19 pandemic situation in order to continue the project, (b) pilot data on the safety of conducting the project through home-based online classes, and (c) the feasibility of ascertaining study outcome measures via videoconferencing.

Trial Modifications

To move the trial forward using an online telehealth approach, it was necessary for the investigative team to make important modifications to our original study protocol outlined previously. These modifications included the following six areas:

1. subject recruitment
2. setting
3. interventions
4. outcome measures
5. assessment
6. data analysis

These modifications, which are described in detail below and are reported in accordance with the current guidelines for completed trials modified due to the COVID-19 pandemic and other extenuating cirumstances,[39] were approved by ORI's IRB on April 2020 and were fully pilot evaluated for feasibility and safety, with results shown in two published studies, one in 2021[40] and one in 2022.[41] No alternations were made to the trial timeline, trial design, or target population (including eligibility criteria and sample size).

1. Modifications made to subject recruitment

Between March and June 2020, due to COVID-19-related restrictions on in-person contacts, all activities related to recruitment were moved online via telephone and Zoom (HIPAA-compliant) videoconferencing. (Note: All modified recruitment activities remained HIPAA compliant). Specific modifications made to study recruitment are described below.

Relevance to the current study: Yes.

Study promotion to identify participants. Our modified recruitment methods involved mainly (a) mass mailing, (b) social media, (c) word of mouth (by telephone or e-mail communications), and (d) digital advisement. Because the online delivery platform of our intervention was no longer constrained by geographic region, we expanded our study sampling area from the originally planned recruitment area in the state of Oregon to include other cities and towns throughout the continental United States (U.S.), as described below.

Study recruitment areas. For the purpose of enhancing generalizability, we purposely focused our recruitment efforts on four geographic regions (Northeast, Midwest, South, and West) in the U.S. and included states that had a high proportion of the population aged 65 years and older (i.e., Maine, Florida, West Virginia, Vermont, Montana). Following the identification of our recruitment regions, special attention was given to targeting counties and cities within each state that, per the US Census Bureau, had the highest index scores on Racial and Ethnic Diversity (www.census.gov/library/stories/state-by-state.html).

Study recruitment procedure. The modified subject contact procedure involved the following:

1. The study recruiter made a return phone call to those who responded to our study promotions. During these calls, a prescreening was conducted with respect to eligibility related to age and memory.
2. Those potential participants who met the initial entry criteria were further screened, via Zoom conferencing, for eligibility, including CDR (both participants and informants) and MMSE.
3. Those who met all eligibility criteria were immediately scheduled for a baseline assessment via Zoom. The assessment was completed by an outcome assessor who was blinded to group allocation.
4. Each participant received an e-mail with a secure (password-protected) ORI Zoom link on the assessment day.
5. On the assessment day, the assessor:
    a. initiated a phone call
    b. described the assessment protocols
    c. visually checked the home environment
    d. conducted the assessment per the protocol

    The assessment was completed by an outcome assessor who was blinded to group allocation.
6. Those who met the study eligibility criteria, signed the study consent form, and completed baseline assessment were randomized into one of the three interventions.

2. Modifications made to intervention setting

The originally planned community-based intervention delivery approach was replaced by hosting the intervention classes online via videoconferencing using Zoom. This switch from in-person class delivery mode to a home-based online delivery mode allowed us to both effectively and efficiently resume our intervention while maintaining the interactive features of in-person, instructor-led training in a face-to-face social context.

Relevance to the current study: Yes.

3. Modifications made to interventions

Prior to resuming the intervention classes, appropriate modifications were made to fit the online videoconferencing delivery method. While the original in-person exercise protocol was used for each intervention group, some minor practical modifications were made to fit the interventions in a home situation. The home-based videoconferencing protocol included the following:

1. Participants received an ORI-initiated Zoom link sent via e-mail, and the exercises were done at home.
2. Instructors delivered the exercise classes from our ORI research facility.
3. Virtual class sessions were delivered twice per week, as originally planned.
4. Each exercise class session was closely supervised and monitored by the project staff for safety and compliance.

Relevance to the current study: Yes.

4. Modifications made to outcome measures

Removal of planned outcome measures. To accommodate the online assessment environment, we had to remove a number of the secondary measures that were planned via the in-person assessment protocol. Removal of the following measures from the assessment protocol were approved by ORI's IRB (dated May 5, 2020):

(a) all computerized cognitive measures (One Card Learning, Block Design, Identification, International Shipping List, and One Back)
(b) Stroop test
(c) Digit Symbol coding
(d) the lab-based *i*TUG (6-meter walk) test involving walking under single-task and dual-task conditions

Modifications to other outcome measures. To accommodate the outcome assessment conducted in a virtual (home) environment, modifications were made to some of the other cognitive and physical performance measures. Details for each are described below. Modifications to the measures were approved by ORI's IRB. These modified measures were pilot evaluated, and results from these evaluations have been published.[12,13]

Montreal Cognitive Assessment (MoCA). In the Visuospatial/Executive section of the MoCA, participants were asked to verbally connect each letter to the corresponding number for the Trail Making task and draw both the object (e.g., cube) and clock on a piece of paper. After they completed the task, participants were asked to show the drawing to the assessor for evaluation. In completing the "Read list of letters" task in the Attention section, the letter-tapping task was replaced by asking participants to count the number of A's in the list.

Trail-Making B (TMT-B). Due to remote operation, this measure was administered verbally. Specifically, the hand-drawing task was replaced by asking participants to verbally recite numbers of letters out loud, alternating between numbers and letters (i.e., 1-A-2-B-3-C, etc.). The study assessor recorded the time needed to complete the task.

Timed Up&Go (TUG). Before each walk, participants were asked to measure or estimate a 10-foot (3-meter) distance away from a table or device (PC or iPad) (see the detail below). The remaining test procedure followed the in-person assessment protocol, that is, participants were asked to (a) stand up from a chair, (b) walk (10 feet forward) at a normal pace to an imaginary straight line on the floor, (c) turn, (d) walk back (10 feet) to the chair, and (e) sit down. The same protocol was applied for the walk under a dual-task condition, where participants were asked to walk while performing an arithmetic task (i.e., starting at the number 81 and sequentially subtracting 3 from the resulting number). No specific verbal instructions were given for prioritization of one of the walking tasks during the dual-task walking trial.

Instructions. Prior to each of the scheduled assessments, participants were informed, by an e-mail, to prepare the following for the upcoming TUG assessment:

a. a measuring tape
b. a piece of paper
c. a standard height chair (without wheels)
d. an open home space of at least 12 feet in length (of which 10 feet will be measured on the floor for the walk test)
e. regular footwear

On the day of the assessment, participants were asked to (a) confirm the 10-foot walkway, (b) place the chair at the end of the walkway (away from the viewing device) and the piece of paper on the floor at the other end of walkway (near the viewing device), and (c) leave 2 extra feet to allow a safe turn around the piece of paper.

The total duration (in seconds) during the 20-feet walk (10 feet away from the chair and 10 feet toward the chair), at normal pace, was recorded for both walking conditions. The dual-task costs, measured in percentage, at each time point were calculated as follows: (dual-task gait speed – single-task

gait speed) / single-task gait speed x 100). The final dual-task costs on gait speed were estimated by taking the difference between dual-task costs at baseline and at 6 months, with negative values indicating deteriorated performance in dual-task walking speed (i.e., dual-task cost), whereas positive values represent an improvement in dual-task walking speed with respect to single-task (i.e., dual-task benefit).

Relevance to the current study: Yes. The in-person survey administration was changed to the online completed via qualtrics^{xm} (qualtrics.com).

5. Modifications made to the assessment protocol

Modified assessment procedure. We modified our follow-up assessment protocol (involving both cognitive and physical performance measures) to accommodate the switch from in-person to online assessment conducted via Zoom. Modifications included the following:

1. The study assessor made a phone call or sent an e-mail reminder to the study participant, notifying the participant of the follow-up assessment and requesting that it be scheduled.
2. Prior to the scheduled assessment date, the participant received an e-mail with a secure ORI Zoom link.
3. On the assessment day, the assessor:
    a. admitted the participant into the Zoom session
    b. described the assessment activities
    c. visually checked the home environment
    d. conducted the assessment per the protocol.

Special note: Because there was no in-person assessment, information about each participant's weight, height, and blood pressure was collected via a self-report during the online assessment.

Modified survey completion procedure. We established a secure online site to allow participants to complete their study surveys online via Qualtrics (Qualtrics.com) if they chose to. Participants could still choose to submit their surveys via regular U.S. mail, as planned in the original protocol.

Relevance to the current study: Yes.

6. Modifications made to data analysis

No major modifications were made from the original statistical plan. However, in order to examine whether the intervention effects were impacted by shifting from in-person (before COVID-19 restrictions) to virtual delivery, we conducted post hoc sensitivity analyses on the two primary outcomes by excluding those who participated in our COVID-19-induced hybrid protocol. The results were reported in our published primary study.

References Cited

1. Li F, Harmer P, Eckstrom E, et al. Clinical effectiveness of cognitively enhanced tai ji quan training on global cognition and dual-task performance during walking in older adults with mild cognitive impairment or self-reported memory concerns: A randomized controlled trial. *Ann Intern Med* 2023;176:1498-1507.
2. Morris JC. The Clinical Dementia Rating (CDR): current version and scoring rules. *Neurol* 1993;43:2412–4.
3. Folstein MF, Folstein SE, McHugh PR. "Mini-mental state": a practical method for grading the cognitive state of patients for the clinician. *J Psychiatr Res* 1975;12(3):189–98.
4. Alden D, Austin CN, Sturgeon R. A correlation between the Geriatric Depression Scale Long and Short Forms. *J Gerontol* 1989;44:124-125.
5. Li F, Harmer P, Fitzgerald K, et al. Effectiveness of a therapeutic Tai Ji Quan intervention vs a multimodal exercise intervention to prevent falls among older adults at high risk of falling: A randomized clinical trial. *JAMA Intern Med* 2018;178(10):1301–1310.
6. Craig, CL, Marshall, AL, Sjostrom, M, Bauman, AE, Booth, ML, Ainsworth, BE, et al. International physical activity questionnaire: 12-country reliability and validity. *Med Sci Sports Exerc* 2003;35:1381–1395.
7. Powell LE, Myers AM. The Activities-specific Balance Confidence (ABC) Scale. *J Gerontology: Med Sci* 1995;50:M28-M34.
8. Podsiadlo D, Richardson S. The timed "Up & Go": a test of basic functional mobility for frail elderly persons. *J Am Geriatr Soc* 1991;39(2):142-148.
9. Kettle VE, Madigan CD, Coombe A, et al. Effectiveness of physical activity interventions delivered or prompted by health professionals in primary care settings: systematic review and meta-analysis of rando missed controlled trials. *BMJ* 2022;376:e068465.
10. 2018 Physical Activity Guidelines Advisory Committee. 2018 Physical Activity Guidelines Advisory Committee Scientific Report. Washington, DC US. Department of Health and Human Services, 2018. Available at: https://health.gov/sites/default/files/2019-09/PAG_Advisory_Committee_Report.pdf. Accessed July 1, 2024.
11. Nasreddine ZS, Phillips NA, Bédirian V, Charbonneau S, Whitehead V, Collin I, Cummings JL, Chertkow H. The Montreal Cognitive Assessment, MoCA: a brief screening tool for mild cognitive impairment. *J Am Geriatr Soc* 2005;53(4):695-9.
12. Li F, Harmer P, Voit J, Chou LS. Implementing an online virtual falls prevention intervention during a public health pandemic for older adults with mild cognitive impairment: A feasibility trial. *Clin Interv Aging* 2021;16:973-983.
13. Li F, Harmer P, Fitzgerald K, Winters-Stone K. A cognitively enhanced, virtual exercise intervention for older adults with mild cognitive impairment: A feasibility trial. BMC *Geriatrics* 2022;22:76.
