# Supplementary material for: Physical Activity Engagement After Tai Ji Quan Intervention Among Older Adults With Mild Cognitive Impairment or Memory Concerns: A Secondary Analysis of a Randomized Clinical Trial
Source: JAMA Netw Open. 2024 Dec 17;7(12):e2450457. doi: 10.1001/jamanetworkopen.2024.50457 (PMC11653122; doi:10.1001/jamanetworkopen.2024.50457)
Supplement: Supplement 2. — eTable 1. Estimated Means on Primary and Secondary Outcomes at Baseline, 4-Month, 6-Month, and 12-Month Follow-Ups and Between-Group Differences in Change From Baseline at 12 Months (Intention-to-Treat Analysis Using Mixed Models)—With Time-Specific Effects eTable 2. Estimated Means on Moderate to Vigorous Intensity Physical Activity at 6-Month and 12-Month Follow-Ups and Within-Group Differences in Change From 6 Months to 12 Months (Intention-to-Treat Analysis Using Mixed Models) eTable 3. Estimated Means on Primary and Secondary Outcomes at Baseline, 4-Month, 6-Month, and 12-Month Follow-Ups and Between-Group Differences in Change From Baseline at 12 Months (Intention-to-Treat Analysis Using Multiple Imputation) eTable 4. Statistical Test Results From the Planned Subgroup Analyses Involving Group by Subgroup (Intervention Compliance, Mobility, Mental Status, and Sex) by Time on Moderate to Vigorous Intensity Physical Activity eAppendix. Psychometrics, Adverse Events, and Data Imputation [file jamanetwopen-e2450457-s002.pdf]

Supplementary Online Content

Li F, Harmer P, Eckstrom E, Winters-Stone K. Physical activity engagement after tai ji quan intervention among older adults with mild cognitive impairment or memory concerns: a secondary analysis of a randomized clinical trial. *JAMA Netw Open*. 2024;7(12):e2450457. doi:10.1001/jamanetworkopen.2024.50457

eTable 1. Estimated Means on Primary and Secondary Outcomes at Baseline, 4-Month, 6-Month, and 12-Month Follow-Ups and Between-Group Differences in Change From Baseline at 12 Months (Intention-to-Treat Analysis Using Mixed Models)—With Time-Specific Effects

eTable 2. Estimated Means on Moderate to Vigorous Intensity Physical Activity at 6-Month and 12-Month Follow-Ups and Within-Group Differences in Change From 6 Months to 12 Months (Intention-to-Treat Analysis Using Mixed Models)

eTable 3. Estimated Means on Primary and Secondary Outcomes at Baseline, 4-Month, 6-Month, and 12-Month Follow-Ups and Between-Group Differences in Change From Baseline at 12 Months (Intention-to-Treat Analysis Using Multiple Imputation)

eTable 4. Statistical Test Results From the Planned Subgroup Analyses Involving Group by Subgroup (Intervention Compliance, Mobility, Mental Status, and Sex) by Time on Moderate to Vigorous Intensity Physical Activity

eAppendix. Psychometrics, Adverse Events, and Data Imputation

This supplementary material has been provided by the authors to give readers additional information about their work.

© 2024 Li F et al. *JAMA Network Open.*

eTable 1. Estimated Means on Primary and Secondary Outcomes at Baseline, 4-Month, 6-Month, and 12-Month Follow-Ups and Between-Group Differences in Change From Baseline at 12 Months (Intention-to-Treat Analysis Using Mixed Models)—With Time-Specific Effects

	Standard tai ji quan (n = 107)	Cognitively enhanced tai ji quan (n = 105)	Stretching exercise (n = 106)	Between-Group Difference in Mean Change from Baseline at 12 Months			
Measure[a]				Standard tai ji quan vs stretching exercise (95% CI)	P value	Cognitively enhanced tai ji quan vs stretching exercise (95% CI)	P value
Moderate and vigorous intensity PA (min/week)							
Baseline	145.4 (12.2)	146.3 (12.3)	145.8 (12.3)				
4 months	232.1 (12.2)	224.8 (12.4)	143.5 (12.4)	89.0 (51.5 – 126.5)	<0.001	80.9 (43.1 – 118.7))	<0.001
6 months	267.9 (12.4)	273.6 (12.5)	147.6 (12.6)	120.75 (79.8 – 161.7)	<0.001	125.5 (84.4 – 166.6)	<0.001
12 months	195.2 (12.5)	195.5 (12.5)	129.4 (12.8)	66.2 (24.5 – 108.0)	0.002	65.6 (23.8 – 107.5)	0.002
Moderate intensity PA (min/week)							
Baseline	110.6 (7.2)	109.4 (7.3)	110.9 (7.2)				
4 months	145.5 (7.3)	142.2 (7.3)	103.1 (7.4)	42.7 (19.9 – 65.6)	<0.001	40.6 (17.6 – 63.7)	0.001
6 months	163.1 (7.2)	163.1 (7.4)	105.6 (7.5)	57.8 (32.9 – 82.8))	<0.001	59.0 (34.0 – 84.0)	<0.001
12 months	131.9 (7.4)	135.0 (7.4)	100.9 (7.6)	31.4 (6.0 – 56.8)	0.02	35.6 (10.2 – 61.1)	0.006
Vigorous intensity PA (min/week)							
Baseline	17.4 (3.9)	18.5 (3.9)	17.5 (3.9)				
4 months	43.3 (3.9)	41.3 (4.0)	20.2 (4.0)	23.0 (10.2 – 10.2 – 35.8)	<0.001	20.0 (7.1 – 32.9)	0.002
6 months	52.5 (3.9)	55.3 (4.0)	21.1 (4.0)	31.4 (17.6 – 45.1)	<0.001	33.1 (19.4 – 46.9)	<0.001
12 months	31.6 (4.0)	30.3 (4.0)	14.4 (4.1)	17.2 (3.3 – 31.2)	0.01	14.9 (1.0 – 28.8)	0.03
Movement Confidence							
Baseline	82.6 (1.3)	82.4 (1.3)	81.6 (1.3)				
4 months	86.6 (1.3)	85.0 (1.3)	82.8 (1.3)	2.8 (-0.1 – 5.7)	0.5	1.4 (-1.5 – 4.3)	0.3
6 months	88.3 (1.3)	89.5 (1.3)	83.1 (1.3)	4.2 (1.0 – 7.3)	0.01	5.6 (2.4 – 8.8)	0.001
12 months	88.4 (1.3)	89.9 (1.3)	82.6 (1.3)	4.9 (1.6 – 8.2)	0.004	6.5 (3.2 – 9.8)	<0.001

Abbreviations: CI, confidence interval. PA, physical activity

[a]Values are means, and values in parentheses are their corresponding standard error of estimates.

© 2024 Li F et al. *JAMA Network Open.*

eTable 2. Estimated Means on Moderate to Vigorous Intensity Physical Activity at 6-Month and 12-Month Follow-Ups and Within-Group Differences in Change From 6 Months to 12 Months (Intention-to-Treat Analysis Using Mixed Models)

			Within-Group Difference Between 6 Months and 12 Months (95% CI)	P Value
Intervention	MVPA (min/week) at 6 Months	MVPA (min/week) at 12 Months		
Standard tai ji quan (n = 107)	267.8 (12.4)	195.2 (12.5)	-72.7 (-109.0 – -36.4)	< 0.001
Cognitively enhanced tai ji quan (n = 105)	273.6 (12.4)	195.5 (12.3)	-78.1 (-114.3 – -41.8)	< 0.001
Stretching exercise (n = 106)	147.6 (12.4)	129.4 (12.2)	-18.2 (-55.4 – 19)	0.9

Abbreviations: MVPA, moderate to vigorous intensity physical activity

The difference score between the time two points (6 Months and 12 Months) was calculated as 12-months score minuses 6-months score.

© 2024 Li F et al. *JAMA Network Open.*

eTable 3. Estimated Means on Primary and Secondary Outcomes at Baseline, 4-Month, 6-Month, and 12-Month Follow-Ups and Between-Group Differences in Change From Baseline at 12 Months (Intention-to-Treat Analysis Using Multiple Imputation)

	Standard tai ji quan (n = 107)	Cognitively enhanced tai ji quan (n = 105)	Stretching exercise (n = 106)	Between-Group Difference in Mean Change from Baseline at 12 Months	
Measure[a]				Standard tai ji quan vs stretching exercise (95% CI), P value	Cognitively enhanced tai ji quan vs stretching exercise (95% CI), P value
Moderate to vigorous intensity PA					
Baseline	145.0 (13.0)	147.4 (13.1)	146.4 (12.2)		
4 months	232.0 (13.5)	226.1 (13.9)	145.2 (12.2)		
6 months	267.9 (13.0)	273.2 (13.1)	149.7 (12.4)	58.4 (20.75 – 96.2)	56.1 (18.4 – 93.8)
12 months	188.2 (10.5)	188.5 (9.8)	131.0 (10.7)	= 0.002	= 0.002
Moderate intensity PA (min/week)					
Baseline	111.0 (8)	110.4 (8)	110.9 (8)		
4 months	145.5 (7)	141.8 (7)	103.4 (7)		
6 months	163.5 (7)	162.6 (8)	107.4 (8)	26.9 (1.2 – 52.8)	29.9 (2.4 – 57.4)
12 months	125.3 (6)	128.2 (6)	98.7 (6)	= 0.001	= 0.002
Vigorous intensity PA (min/week)					
Baseline	17.4 (3)	19.0 (3)	17.5 (3)		
4 months	43.7 (5)	41.4 (5)	20.6 (5)		
6 months	52.8 (5)	55.9 (5)	24.8 (5)	14.4 (3.2 – 25.5)	11.4 (1.0 – 21.8)
12 months	32.2 (3)	30.8 (3)	17.9 (3)	= 0.01	= 0.004
Movement Confidence					
Baseline	82.6 (1.1)	82.4 (1.1)	81.6 (1.1)		

© 2024 Li F et al. *JAMA Network Open.*

4 months	86.6 (1.5)	84.9 (1.5)	82.8 (1.5)		
6 months	88.3 (1.2)	89.4 (1.2)	83.2 (1.2)	4.6 (1.4 – 7.8)	6.2 (2.9 – 9.3)
12 months	88.4 (1.2)	89.8 (1.2)	82.8 (1.2)	= 0.002	<0.001

Abbreviations: CI, confidence interval. PA, physical activity

[a]Values are means, and values in parentheses are their corresponding standard error of estimates.

© 2024 Li F et al. *JAMA Network Open.*

eTable 4. Statistical Test Results From the Planned Subgroup Analyses Involving Group by Subgroup (Intervention Compliance, Mobility, Mental Status, and Sex) by Time on Moderate to Vigorous Intensity Physical Activity

**Measure**	**F statistic**	**P value**
MVPA[a]		
Group by Intervention Compliance[b] by Time	1.2 (12, 459)	0.3
Group by mobility[c] by Time	1.6 (12, 424)	0.9
Group by mental status[d] by Time	1.0 (12, 420)	0.4
Group by Sex by Time	1.3 (12, 426)	0.2

[a] Moderate to Vigorous Intensity Physical Activity
[b]Defined intervention class attendance: adherers ((≥75% of exercise class attendance)
[c]Defined by Timed Up&Go test: impaired (≥12 seconds) vs nonimpaired (<12 seconds)
[d]Defined by Mini-Mental State Examination: cognitively impaired (≤27) vs intact (>27)

© 2024 Li F et al. *JAMA Network Open.*

eAppendix. Psychometrics, Adverse Events, and Data Imputation

Data on the psychometrics of the International Physical Activity Questionnaire (related to this trial study)

Reliability

- Test-retest (4-months interval) reliability estimates from this study: α 0.89 (unpublished data from this study)
- Test-retest (4-months interval) reliability estimates from this study: α 0.87 (data from Li et al. 2018 study)

Validity

- Concurrent validity: MVPA of IPAQ was moderately correlated with Physical Activity Scale for the Elderly (PASE), r = 0.78 (data from Li et al., 2018 study)
- Criterion validity: a moderate correlation between walking steps, measured by pedometers, and MVPA measured by IPAQ (r = 0.59) (unpublished data from a pilot study of this large study)

Adverse Events

There were 38 adverse events documented from this trial. Seven serious adverse events required hospitalization, but none were deemed intervention related. Nine moderate adverse events required medical surgeries/visits and were also not related to the intervention. Of the 22 mild adverse events reported, 6 were possibly intervention related and 3 were definitely intervention related, mainly exercise-induced muscle soreness or pain.

Data Imputation

In a sensitivity analysis, we used a multiple imputation data method (chained equations in SPSS) to impute missing data across all time points on both the primary and secondary outcomes ascertained from the study participants. This missing imputation approach assumes that data are missing at random or are missing completely at random. We performed 10 sets of imputations. Variables without missing data were used for prediction of imputed values on the study variables. These included baseline measures of age, sex, level of education, MMSE, number of chronic conditions, and depression (see SPSS syntax below). The imputed data were then submitted to the same analytic model for analysis (i.e., linear mixed model) as the non-imputed observed data. The results of each analytic model were pooled across the 10 imputed data sets.

```
*Impute Missing Data Values.
DATASET DECLARE MVPA_impute_10.
MULTIPLE IMPUTATION ayrr sex mmse dep1 educat chronir mvpa1 mvpa2 mvpa3 mvpa4
  /IMPUTE METHOD=AUTO NIMPUTATIONS=10 MAXPCTMISSING=NONE
  /MISSINGSUMMARIES NONE
  /IMPUTATIONSUMMARIES MODELS DESCRIPTIVES
  /OUTFILE IMPUTATIONS=MVPA_impute_10.
```

© 2024 Li F et al. *JAMA Network Open.*
