# Supplementary material for: Physical Activity Engagement After Tai Ji Quan Intervention Among Older Adults With Mild Cognitive Impairment or Memory Concerns: A Secondary Analysis of a Randomized Clinical Trial
Source: JAMA Netw Open. 2024 Dec 17;7(12):e2450457. doi: 10.1001/jamanetworkopen.2024.50457 (PMC11653122; doi:10.1001/jamanetworkopen.2024.50457)
Supplement: Supplement 3. — Data Sharing Statement [file jamanetwopen-e2450457-s003.pdf]

Data Sharing Statement

Li. Physical Activity Engagement After Tai Ji Quan Intervention Among Older Adults With Mild Cognitive Impairment or Memory Concerns. *JAMA Netw Open*. Published December 17, 2024. doi:10.1001/jamanetworkopen.2024.50457

Data
Additional Information: Clinicaltrials.gov Identifier: NCT04070703
Data available: Yes
Data types: Deidentified participant data
How to access data: fuzhongl@ori.org
When available: With publication

Supporting Documents
Document types: Statistical/analytic code
How to access documents: fuzhongl@ori.org
When available: With publication

Additional Information
Who can access the data: researchers whose proposed use of the data has been approved
Types of analyses: physical activity
Mechanisms of data availability: after approval of a proposal
